# Non-adherence to antihypertensive drugs and its risk factors among hypertensive patients, Marrakech, Morocco

**DOI:** 10.1371/journal.pgph.0002774

**Published:** 2024-08-27

**Authors:** Safae Belayachi, Fatima Zahra Boukhari, Firdaous Essayagh, Othmane Terkiba, Alban Zohoun, Meriem Essayagh, Touria Essayagh, Sanah Essayagh

**Affiliations:** 1 Laboratoire Agroalimentaire et Santé, Faculté des Sciences et Techniques, Hassan First University of Settat, Settat, Morocco; 2 Laboratoire Droit Privé et Enjeux de Développement, Faculté des Sciences Juridiques, Économiques et Sociales, Université Sidi Mohamed Ben Abdellah, Fès, Morocco; 3 Laboratoire Sciences et Technologies de la Santé, Institut Supérieur des Sciences de la Santé, Hassan First University of Settat, Settat, Morocco; 4 Unité D’enseignement et de Recherche en Hématologie, Faculté des Sciences de la Santé, Université d’Abomey-Calavi, Cotonou, Bénin; 5 Office National de Sécurité Sanitaire des Produits Alimentaires, Oriental, Morocco; Augusta University, UNITED STATES OF AMERICA

## Abstract

Non-adherence to hypertensive drugs is a barrier to controlling blood pressure and decreases hypertensive patients’ quality of life. The aim of this study was to determine the prevalence of non-adherence to hypertensive drugs among hypertensive patients treated at Marrakech’s primary health care facilities. A cross-sectional survey of 922 hypertensive patients treated at Marrakech’s primary health care facilities for arterial hypertension was conducted between 2021 and 2022. For collecting data, two questionnaires were employed. One was administered during an interview to patients and focused on socio-demographic, behavioral, and clinical variables, as well as hypertensive treatment characteristics and the care-patient-physician triad. The physician self-administered the second questionnaire to assess therapeutic inertia. Non-adherence risk factors were identified using multivariate logistic regression. A total of 760 participants did not adhere to the hypertensive drugs, with a prevalence of 82.4%. The average age was 62.8±9.8 years, and 600 (78.9%) of the participants were female. Moderate stress, unsatisfactory family support, uncontrolled hypertension, the presence of depressive symptoms, an insufficient patient-physician interaction, and inadequate medical management of cardiovascular risk factors were associated with drug non-adherence. Non-adherence to hypertensive treatment is common in Marrakech. Regular therapeutic education classes and support group meetings must be scheduled. A performance-based remuneration system to incentivize health-care workers should be investigated.

## Introduction

High blood pressure (HBP) is a public health issue that kills 9.4 million people each year [1]. Worldwide, in 2023, 1.28 billion people aged 30–79 years were estimated to have hypertension, with two-thirds living in low- and middle-income countries. According to the most recent national survey on the risk factors for cardiovascular disease, which was done among a sample of 5429 adults in Morocco in 2017, the prevalence of hypertension was 29.3% [2].

The objective of hypertension therapeutic management is to control blood pressure, which is not always easy in practice, and hypertension remains uncontrolled [3]. The study conducted in Meknes in 2017 on a sample of 922 people showed a prevalence of uncontrolled blood pressure of 73% [4].

The scientific literature indicates a beneficial relationship between hypertensive drugs and blood pressure control [5, 6]. However, drugs can only help lower blood pressure if the patients participate in the management and follow the physician’s therapeutic recommendations [7].

Non-adherence to hypertensive drugs, defined as a patient’s inability to follow medical instructions about medication dosage, time, and frequency, is a risk factor for uncontrolled blood pressure [6]. Non-adherence to hypertensive drugs has been linked to uncontrolled blood pressure, which can result in cardiovascular diseases, stroke, chronic renal failure, disability, and premature death.

The literature reports that depression could have an impact on the cognitive concentration and motivation of patients. It could even affect their desire and ability to follow the prescriptions of treating physicians [8]. Epidemiological studies had shown that a higher percentage of non-adherence was observed in hypertensive patients presenting symptoms of depression [9, 10].

A study including 254 patients in Ghana found a prevalence of non-adherence to hypertensive drugs of 63.7% [7]. Out of 922 hypertensive patients observed at basic health care institutions in Meknes, Morocco, the prevalence of non-adherence to hypertensive drugs was 91.0% [6]. In the Meknes study, several variables were treated but without assessing the impact of depression on adherence to antihypertensive drugs.

Non-adherence to hypertensive drugs is a barrier to achieving sustainable development goals in terms of population health as well as a loss of resources for the health system. It contributes to a decrease in citizens’ trust in the national health system. The goal of our study was to determine the prevalence of non-adherence to hypertensive drugs among hypertensive patients treated at primary health care facilities in Marrakech, as well as the risk factors associated with it.

It’s important to mentioned that the same study had been conducted in Meknes, Morocco by the same group and the current study is a continuation of that research. Furthermore, the study methods were similar in the two sites with the exception of this study in Marrakech where self-reported depression was assessed.

## Methods

Marrakech is a city in central Morocco, close to the Atlas Mountains. It has a population of 1,330,468 people and an area of around 2625 km2. In terms of population, Marrakech is the country’s third-largest agglomeration [11]. It includes 23,213 hypertension patients treated in primary care facilities [12].

We conducted a cross-sectional study on hypertensive patients treated at primary health care facilities in Marrakech between 26^th^ May 2021 and 30^th^ December 2022. A two-stage stratified survey was used for sampling. 70% of hypertensives lived in urban areas, while 30% lived in rural areas. The delegation from Marrakech’s Ministry of Health and Social Protection offered a thorough list of primary health care facilities. A first draw was held to determine the primary unit, which consists of primary health care facilities. The secondary unit, which was made up of hypertensive individuals, was determined by a second draw. On the day of the survey, hypertensive patients who arrived at the primary health care facility were given a queue number, and these numbers were selected at random to choose individuals until the desired sample size was attained. We only included in the study hypertensive patients who had been following the Marrakech Primary Health Care facilities for at least six months and had accepted to participate in the study. Pregnant women and people with mental illnesses were excluded from the trial.

### Sample size

Our sample size was limited to 922 patients. With a confidence range of 95%, a margin of error of 5%, and a non-response rate of 20%, the estimated prevalence of drugs non-adherence was 50%.

### Data collection tool and procedure

Data collection was carried out through two questionnaires. The first questionnaire was administered face-to-face after interviewing the patients. This questionnaire includes socio-demographic and economic data, behavioral characteristics, hypertension knowledge, clinical characteristics, hypertensive treatment characteristics, and characteristics of the relationship between the care system, patient, and physician. The second questionnaire, which assessed therapeutic inertia, was self-administered by the attending physician.

### Operational definitions

#### Assessment of drug non-adherence

The Gired assessment test was used to assess non-adherence to hypertensive drugs. This test contains six questions to which the patient has to answer "yes" or "no". These questions are: (i) Did you forget to take your drug this morning? (ii) Since the last consultation, have you been out of drugs? (iii) Since the last consultation, have you ever taken your treatment later than usual? (iv) Since the last consultation, have you ever failed to take your drug because, on certain days, your memory fails you? (v) Since the last consultation, have you ever failed to take your drug because, on some days, you felt that it was doing you more harm than good? (vi) Since the last consultation, do you think you have too many tablets to take? If the score for "yes" answers was greater than or equal to one, we spoke of drug non-adherence. When there was no "yes" answer, we were talking about drug adherence [13].

#### Blood pressure measurement

An electronic tensiometer with an adjustable cuff (MicroLife Pro M with an accuracy of 3 mmHg) was used to monitor blood pressure (BP). Blood pressure control was evaluated using the criteria of the European Society of Arterial Hypertension and the European Society of Cardiology (ESH/ESC) recommendations [14].

#### Therapeutic inertia

When there was no change in treatment despite an uncontrolled blood pressure value during two consecutive visits and a minimum of one month between these two consultations with no change in treatment in the previous three months, therapeutic inertia was declared. Three months is the minimal follow-up duration for a patient with well-controlled hypertension.

#### Assessment of knowledge about high blood pressure

We assessed knowledge about hypertension through a 21-item questionnaire to which the participant had to answer “yes” or “no”. These elements have been grouped into three subgroups: nine questions on symptoms of high blood pressure, four questions on complications, and eight questions on preventive measures. If the participant answered “no” to half of the questions in each subgroup, their knowledge about high blood pressure was rated as unsatisfactory. Conversely, when the participant answered “yes” to half of the questions in each subgroup, he was considered to have “sufficient knowledge about high blood pressure.” The 21 questions were: (i) Is headache a symptom of high blood pressure, (ii) Is an auditory whistling a symptom of high blood pressure, (iii) Is a blurred vision (feeling of flies in front of the eyes)?, (iv) Is dizziness a symptom of high blood pressure?, (v) Is a fast heart rate a symptom of high blood pressure?, (vi) Is a difficulty breathing a symptom of high blood pressure?, (vii) Is a bleeding nose a symptom of high blood pressure?, (iix) Is a presence of blood in the urine a symptom of high blood pressure?, (iix) Is a oedema a symptom of high blood pressure?, (x) Is a stroke a complication of high blood pressure?, (xi) Is a heart attack a complication of high blood pressure?, (xii) Is a kidney damage a complication of high blood pressure?, (xiii) Is a eye damage a complication of high blood pressure?, (ivx) Is a loss of weight a preventive measure against of high blood pressure?, (xv) Is a reduction of a consumption of alcohol a preventive measure against of high blood pressure?, (xvi) Is a reduction of a consumption of tobacco a preventive measure against of high blood pressure?, (xvii) Is a physical activity a preventive measure against of high blood pressure?, (xviii) Is a stress avoidance a preventive measure against of high blood pressure?, (ixx) Is a dietary compliance a preventive measure against of high blood pressure?, (xx) Is a compliance with treatment a preventive measure against of high blood pressure?, (xxi) Self-measurement of blood pressure and regular medical (monitoring) follow-up is a preventive measure against of high blood pressure?.

The Cronbach’s alpha of the instrument was 0.87, indicating satisfactory internal consistency for the hypertension knowledge assessment questionnaire.

Stress was reported when patients reported being stressed. Lack of family support was reported when patients reported having no family support, and symptoms of depression were assessed using the Patient Health Questionnaire-9 (PHQ-9) [15].

Failure to manage cardiovascular risk factors was reported when the physicians declared that they did not manage these factors.

#### Ethical consideration

The Helsinki Declaration was followed throughout the study. After being told about the study’s objectives and having their privacy and confidentiality protected, all participants provided an informed written statement of consent. The study protocol was evaluated and approved by Rabat’s Faculty of Medicine and Pharmacy’s ethical committee (#25/21).

### Statistical analysis

The data were analyzed on Epi-Info version 7. To characterize the data, a descriptive analysis was undertaken. Where applicable, continuous variables were presented as the mean and standard deviation. Numbers and percentages were used to express categorical variables. Categorical variables were compared using the Pearson chi-square test or, where applicable, Fisher’s exact test. Continuous variables were compared using the ANOVA test or the Mann-Whitney test, where appropriate. We included in the multiple logistic regression all variables with a p-value up to 0.20 in the bivariate analysis. The association between the risk factor and non-adherence to hypertensive drugs was determined by the adjusted odds ratio (AOR) and its 95% confidence interval.

## Results

### Socio-demographic and economic characteristics

The socio-demographic and economic characteristics of hypertensive patients are summarized in Table 1. A total of 922 patients were recruited, with an 82.4% non-adherence prevalence to hypertensive drugs. The average age of the participants was 63.1±9.8 years, with extremes ranging from 33 to 102 years, and 760 (78.8%) were females. 281 (30.5%) of participants had a monthly income of less than $150, and 156 (16.9%) did not have health insurance.

**Table 1:** Socio-demographic and economic characteristics of hypertensives followed in primary health care facilities, Marrakech, 2021–2022.

	All participants n (%)	Drug non-adherence n (%)	Drug adherence n (%)
Total participants	922 (100)	760 (82.4)	162 (17.6)
Mean age in years±sd	63.1±9.8	62.8±9.8	64.2±9.9
Sex			
Female	727 (78.8)	600 (78.9)	127 (78.4)
Male	195 (21.2)	160 (21.1)	35 (21.6)
Age group in years			
≥80	54 (05.9)	40 (05.3)	14 (08.6)
[70–79]	182 (19.7)	150 (19.5)	32 (19.7)
[60–69]	386 (41.9)	319 (42.0)	67 (41.4)
[50–59]	225 (24.4)	191 (25.2)	34 (21.0)
Less than or equal to 49	75 (08.1)	60 (08.0)	15 (09.3)
Marital status			
Single	346 (37.5)	284 (37.4)	62 (38.3)
Partnered	576 (62.5)	476 (62.6)	100 (61.7)
Education			
Illiterate	690 (74.8)	560 (73.7)	130 (80.2)
Elementary	151 (16.4)	130 (17.1)	21 (13.0)
Middle school	47 (05.1)	41 (05.4)	6 (03.7)
High school	21 (02.3)	19 (02.5)	2 (01.2)
College	13 (01.4)	10 (01.3)	3 (01.9)
Occupation			
No	833 (90.4)	683 (89.9)	150 (92.6)
Yes	89 (09.6)	77 (10.1)	12 (07.4)
Monthly income ($)			
<150	281 (30.5)	246 (32.3)	35 (21.6)
150–199	368 (39.9)	285 (37.5)	83 (51.2)
200–299	235 (25.5)	199 (26.2)	36 (22.2)
300–499	23 (02.5)	18 (02.4)	5 (03.1)
≥500	15 (01.6)	12 (01.6)	3 (01.9)
Health insurance			
No	156 (16.9)	135 (17.8)	21 (13.0)
Yes	766 (83.1)	625 (82.2)	141 (87.0)

sd: standard deviation

### Behavioral characteristics

Of the 922 participants, 17 (1.8%) had satisfactory general knowledge of hypertension. The distribution of hypertension knowledge levels revealed that 18 (01.9%) had a satisfactory level of knowledge on the signs of the disease, 330 (35.8%) on its complications, and 10 (01.1%) on its prevention measures. 586 (63.6%) participants reported moderate stress, whereas 351 (38.1%) reported no family support “Table 2”.

**Table 2:** Behavioral characteristics of hypertensives followed in primary health care facilities, Marrakech, 2021–2022.

	All participants n = 922	Drug non-adherence n = 760	Drug adherence n = 162
General knowledge about high blood Pressure			
Unsatisfactory	905 (98.2)	750 (98.7)	155 (95.7)
Satisfactory	17 (1.8)	10 (01.3)	7 (04.3)
Knowledge of the signs of high blood pressure			
Unsatisfactory	904 (98.1)	747 (98.3)	157 (96.9)
Satisfactory	18 (01.9)	13 (01.7)	5 (03.1)
Knowledge of complications of high blood pressure			
Unsatisfactory	592 (64.2)	491 (64.6)	101 (62.4)
Satisfactory	330 (35.8)	269 (35.4)	61 (37.6)
Knowledge about preventing measures against high blood pressure			
Unsatisfactory	912 (98.9)	755 (99.3)	157 (96.9)
Satisfactory	10 (01.1)	5 (00.7)	5 (03.1)
Tobacco consumption			
Yes	86 (09.3)	70 (09.2)	16 (09.9)
No	836 (90.7)	690 (90.8)	146 (90.1)
Alcohol consumption			
Yes	39 (04.2)	31 (04.1)	8 (04.9)
No	883 (95.8)	729 (95.9)	154 (95.1)
Stress			
Intense	229 (24.8)	191 (25.1)	38 (23.5)
Intermediate	586 (63.6)	495 (65.1)	91 (56.2)
None	107 (11.6)	74 (09.8)	33 (20.3)
Family support			
No	351 (38.1)	309 (40.7)	42 (25.9)
Yes	571 (61.9)	451 (59.3)	120 (74.1)

### Clinical characteristics

According to the data in Table 3, 464 (50.3%) of the participants had comorbidities, 418 (45.3%) had diabetes, 60 (06.5%) experienced hypertension complications, and 678 (73.5%) had uncontrolled hypertension. Depression symptoms were reported by 551 (59.8%) of the participants.

**Table 3:** The clinical characteristics of hypertensives followed in primary health care facilities, Marrakech, 2021–2022.

	All participants n = 922	Drug non-adherence n = 760	Drug adherence n = 162
Co-morbidities			
Yes	464 (50.3)	365 (48.0)	99 (61.1)
No	458 (49.7)	395 (52.0)	63 (38.9)
Cardiovascular diseases			
Yes	34 (03.7)	25 (03.3)	9 (05.6)
No	888 (96.3)	735 (96.7)	153 (94.4)
Diabetes			
Yes	418 (45.3)	327 (43.0)	91 (56.2)
No	504 (54.7)	433 (57.0)	71 (43.8)
Dyslipidemia			
Yes	54 (05.9)	43 (05.7)	11 (06.8)
No	868 (94.1)	717 (94.3)	151 (93.2)
Presence of complication			
Yes	60 (06.5)	45 (05.9)	15 (09.3)
No	862 (93.5)	715 (94.1)	147 (90.7)
Control of blood pressure			
No	678 (73.5)	576 (75.8)	102 (63.0)
Yes	244 (26.5)	184 (24.2)	60 (37.0)
Family history of high blood pressure			
Yes	446 (48.4)	363 (47.8)	83 (51.2)
No	476 (51.6)	396 (52.2)	79 (48.8)
Symptoms of depression			
Yes	551 (59.8)	482 (63.4)	69 (42.6)
No	371 (40.2)	278 (36.6)	93 (57.4)
Overweight/obesity			
Yes	742 (80.5)	612 (80.5)	130 (80.3)
No	180 (19.5)	148 (19.5)	32 (19.7)

### Characteristics related to treatment and the patient-physician-healthcare system triad

Of the 922 participants, 146 (15.8%) declared they had a good relationship with the healthcare system, 144 (15.6%) declared they had a good relationship with their physician, 444 (48.2%) declared they had medical management for cardiovascular risk factors, and 878 (95.2%) were on triple therapy. Therapeutic inertia was 42.3% “Table 4”.

**Table 4:** Characteristics of drug treatment and the Patient-Doctor-Healthcare system triad, Marrakech, 2021–2022.

	All participants n = 922	Drug non-adherence n = 760	Drug adherence n = 162
Relationship with the healthcare system			
Unsatisfactory	776 (84.2)	651 (85.7)	125 (77.2)
Satisfactory	146 (15.8)	109 (14.3)	37 (22.8)
Physician–patient relationship			
Unsatisfactory	778 (84.4)	652 (85.8)	126 (77.8)
Satisfactory	144 (15.6)	108 (14.2)	36 (22.2)
Medical management of cardiovascular risk factors			
No	478 (51.8)	413 (54.3)	65 (40.1)
Yes	444 (48.2)	347 (45.7)	97 (59.9)
Therapeutic inertia (n = 892)			
Yes	377 (42.3)	317 (43.3)	60 (37.5)
No	515 (57.7)	415 (56.7)	100 (62.5)
Traitement			
Triple therapy	878 (95.2)	722 (95.0)	156 (96.3)
Dual therapy	38 (04.1)	33 (04.3)	5 (03.1)
Monotherapy	6 (00.7)	5 (00.7)	1 (00.6)
Duration of the last hypertensive medication in a month			
More than six months	446 (48.4)	363 (47.8)	83 (51.2)
≤ six months	476 (51.6)	397 (52.2)	79 (48.8)

### Non-adherence to hypertensive drugs

Among 760 hypertensive patients with non-adherence to antihypertensive drug, 696 (91.6%) declared taking their drugs late compared to the usual time, 425 (55.9%) declared not having taken their drugs because on certain days their memory failed them, 355 (46.7%) declared that they had forgotten to take their drugs on the day of the survey, 247 (32.5%) stated that they did not take antihypertensive drugs following a discontinued medication since the last consultation, and 243 (32.0%) reported not taking antihypertensive drugs because on certain days they felt that the treatment was doing them more harm than good “Table 5”.

**Table 5:** Distribution of non-adherence to antihypertensive drug, Marrakech, 2021–2022.

	All participants n = 922	Drug non-adherence n = 760	Drug adherence n = 162
Having forget to take your drug this morning			
Yes	355 (38.5)	355 (46.7)	0 (00.0)
No	567 (61.5)	405 (53.3)	162 (100.0)
Being out of drug since the last consultation			
Yes	247 (26.8)	247 (32.5)	0 (00.0)
No	675 (73.2)	513 (67.5)	162 (100.0)
Having to take drug late compared to the usual time			
Yes	696 (75.5)	696 (91.6)	0 (00.0)
No	226 (24.5)	64 (08.4)	162 (100.0)
Having missed drug because some days memory lacking			
Yes	425 (46.1)	425 (55.9)	0 (00.0)
No	497 (53.9)	335 (44.1)	162 (100.0)
Missing a drug because some days you feel like your drug is doing you more harm than good			
Yes	243 (26.4)	243 (32.0)	0 (00.0)
No	679 (73.6)	517 (68.0)	162 (100.0)
Thinking to have too many pills to take			
Yes	54 (05.9)	54 (07.1)	0 (00.0)
No	868 (94.1)	706 (92.9)	162 (100.0)

During the bivariable analysis, the p-value was set at 0.20. As mentioned in Table 6, after bivariable analysis, 17 factors were associated with the presence of non-adherence to hypertensive drugs: (i) age (p-value = 0.12); (ii) low monthly income per household (p-value 0.01); (iii) lack of health insurance (p-value = 0.14); (iv) presence of comorbidities (p-value = 0.002); (v) presence of cardiovascular disease (p-value = 0.16); (vi) presence of diabetes (p-value = 0.002); (vii) presence of complication (p-value = 0.12); (ix) unsatisfactory general knowledge of hypertension (p-value = 0.014); (x) unsatisfactory knowledge about prevention measures (p-value = 0.01); (xi) presence of stress (p-value = 0.001); (xii) unsatisfactory family support (p-value = 0.0005); (xiii) uncontrolled hypertension (p-value = 0.0009); (xiv) presence of symptoms of depression (p-value = <0.0001); (xv) unsatisfactory relationship between patient and healthcare system (p-value = 0.007); (xvi) unsatisfactory patient-physician relationship (p-value = 0.01); and (xvii) lack of medical management of cardiovascular risk factors (p-value = 0.001) “Table 6”.

**Table 6:** Multivariable analysis (odds ratio, p-value) of risk factors associated with drug non-adherence in hypertensive patients, Marrakech, 2021–2022.

	Bivariable analysis		Multivariable analysis complete model	
	COR (95% CI)	*p-value*	AOR (95% CI)	*p-value*
Age in years	0.98 [0.96–1.00]	0.12	0.98 [0.97–11.00]	0.29
Monthly income ($)		0.01		
<150/≥500	1.75 [0.47–6.53]	0.40	0.95 [0.19–4.70]	0.95
150-199/≥500	0.85 [0.23–3.11]	0.81	0.50 [0.10–2.42]	0.39
200-299/≥500	1.38 [0.37–5.14]	0.62	1.00 [0.20–4.83]	0.99
300-499/≥500	0.90 [0.18–4.48]	0.89	0.59 [0.09–3.72]	0.58
Lack of health insurance	1.44 [0.88–2.37]	0.14	1.46 [0.85–2.50]	0.16
Presence of comorbidity	0.58 [0.41–0.83]	0.002	0.96 [0.34–2.75]	0.95
Presence of cardiovascular disease	0.57 [0.26–1.26]	0.16	0.60 [0.22–1.59]	0.30
Presence of diabetes	0.58 [0.41–0.82]	0.002	0.82 [0.29–2.31]	0.71
Presence of complication	0.61 [0.33–1.13]	0.12	0.70 [0.33–1.47]	0.34
General unsatisfactory knowledge of hypertension	3.38 [1.26–9.03]	0.014	0.86 [0.13–5.40]	0.87
Insufficient knowledge about prevention measures against hypertension	4.80 [1.37–16.8]	0.01	5.69 [0.54–60.0]	0.14
Stress		0.001		
Intense/none	2.24 [1.30–3.83]	0.003	1.30 [0.68–2.46]	0.41
Intermediate/none	2.42 [1.52–3.87]	0.0002	1.87 [1.12–3.14]	0.01
Insufficient family support	1.95 [1.33–2.86]	0.0005	1.56 [1.01–2.41]	0.04
Uncontrolled blood pressure	1.84 [1.28–2.63]	0.0009	1.84 [1.14–2.98]	0.01
Symptoms of depression	2.33 [1.65–3.29]	<0.00001	2.35 [1.55–3.56]	0.0001
Insufficient relationship with the healthcare system	1.76 [1.16–2.68]	0.007	1.25 [0.78–2.01]	0.33
Insufficient physician–patient relationship	1.72 [1.13–2.63]	0.01	1.63 [1.01–2.63]	0.04
Insufficient of medical management of risk factors	1.77 [1.25–2.50]	0.001	2.24 [1.12–4.48]	0.02
Therapeutic inertia	1.27 [0.89–1.81]	0.17	0.92 [0.58–1.47]	0.74

COR Crude odds ratio, AOR Adjusted odds ratio, CI Confidence interval

### Multivariable analysis

After adjusting for the other variables, we identified the following factors as being associated with hypertensive drug non-adherence: (i) moderate stress compared to low stress (Adjusted Odd Ratio of 1.87; 95% CI [1.12–3.14]); (ii) lack of family support (AOR of 1.95; 95% CI [1.33–2.86]); (iii) uncontrolled blood pressure (AOR of 1.84; 95% CI [1.14–2.98]); (iv) presence of symptoms of depression (AOR of 2.35; 95% CI [1.55–3.56]); (v) unsatisfactory patient-physician relationship (AOR of 1.63; 95% CI [1.01–2.63]); and (vi) lack of medical management of cardiovascular risk factors (AOR of 2.24; 95% CI [1.12–4.48]) “Table 6”.

## Discussion

The prevalence of non-adherence to hypertensive drugs in hypertensive patients followed at primary health care facilities Marrakech was 82.4%. This result is lower than the prevalence of 91% reported in Meknes, Morocco, in 2017 on a sample of 922 patients [6] and 87.5% reported in Ivory Coast in 2006 on a sample of 200 patients [16]. However, it remains higher than the 64.5% observed in Algeria in 2013 in 453 patients [17] and the 63.4% observed in Sfax, Tunisia, in 2010 in a sample of 273 patients [18].

In France, between 2012 and 2015, in 87,700 hypertensive patients, age was a risk factor associated with non-adherence with hypertensive drugs [19]. The literature associates this with the deterioration of physical and mental functions with age, denial of the disease, the difficulties encountered by elderly subjects in understanding the medical prescriptions of their physicians, the high number of comorbidities and drugs to take every day, difficulties remembering the type of medicine to take or even forgetting to take it, and the undesirable effects that a hypertensive drug can have [20]. Indeed, since hypertension is a disease without symptoms and the benefits of treatment are "silent", the fact that it can develop undesirable effects can discourage people and make them think that the treatment brings more disadvantages than benefits. In our study, age was not associated with non-adherence to hypertensive drugs. This same result was reported in the Meknes study [6].

The literature, including the similar study conducted in Meknes reports low income as a factor associated with non-adherence to hypertensive drugs [6, 16]. In Indeed, although the hypertensive treatment is distributed free of charge to hypertensive patients at the level of the primary health care facilities, households with low monthly incomes, in the event of a shortage of hypertensive drugs at the level of the primary health care facilities, have difficulty obtaining this drug. In our study, low monthly income was not associated with non-adherence to hypertensive drugs.

Lack of access to basic health coverage and the burden of out-of-pocket expenses are important causes of non-adherence to hypertensive drugs. Some people don’t buy hypertensive drugs; others skip daily doses to save money [21]. The study conducted in Quebec in 2014 showed that one in ten Canadians does not respect medical prescriptions because of the price, and 31.3% of the original prescriptions had not been filled after nine months. The fact that the drugs and the costs to be paid by the person are high increases the risk of non-adherence [22]. In our work, the absence of health insurance was not associated with drug non-adherence. This same result was reported in the Meknes study [6].

In the present investigation, being exposed to stress is a risk factor associated with the presence of non-adherence to drugs in hypertensive patients. Indeed, stress can lead to a deterioration of the emotional state and an elevation of anxiety [23]. It is linked to organizational difficulties and a decrease in the level of concentration, which can lead to forgetfulness and interruptions of hypertensive treatment [24]. A stressed person can easily miss medication schedules, may miss prescribed doses, and may not refill prescriptions on a regular basis [25].

Family support has an important role in medication adherence in hypertensives [26]. It helps to emphasize the importance of following a treatment plan and provides suggestions for dealing with problems. It is a means of motivation and encouragement on a daily basis. It allows patients to remain committed to taking medication regularly by avoiding oversights or interruptions and by participating in its purchase in the event of a stock shortage at the primary health care facilities. In our study, insufficient family support was associated with non-adherence to hypertensive drugs. A different result was observed in the Meknes study [6].

In our work, the presence of symptoms of depression characterized by sadness, loss of interest or pleasure, feelings of guilt or low self-esteem, sleep or appetite disturbances, feelings of fatigue, and a lack of concentration have been associated with the presence of non-adherence to hypertensive drugs. This could be explained by the altered cognitive state [27], the unsatisfactory results of the treatment of hypertension, the poorer quality of life, and the high costs for the patients in the management of their chronic disease, which requires medical care and biological assessments. To which is added dissatisfaction with the health system. Failure to meet these needs due to limited resources can exacerbate their health problems [28].

The non-control of the arterial hypertension in spite of the consumption of the hypertensive drug would have a negative influence on the regular follow-up of the drug treatment. This would lead to demotivation of the patient and a loss of confidence in the prescribed hypertensive drug, which increases the prevalence of non-adherence [29]. In our study, uncontrolled blood pressure was associated with drug non-adherence. This same result was reported in the Meknes study [6].

In our study, insufficient medical management of cardiovascular risk factors was associated with non-adherence to hypertensive drugs in patients with hypertension. Indeed, hypertensive patients are subject to cardiovascular risk factors, mainly diabetes, overweight and obesity, tobacco and alcohol consumption, and excessive salt consumption. Failure to take care of these factors would lead to weariness, demotivation, and a lack of perception of the beneficial impact of the treatment, the positive effects of which will only be felt with delay, while the undesirable effects are immediate for the patient and would lead to non-adherence to hypertensive drugs. A different result was observed in the Meknes study [6].

In our survey, an unsatisfactory physician-patient relationship was associated with non-adherence to hypertensive drugs. The literature reports that the relationship between the physician and the patient in the development, implementation, and therapeutic follow-up of patients. The prescription of the drug is a relational act between the physician and the patients, and the quality of this relationship and the trust that patients have in their physician influence their adherence. People are more likely to adhere to medication if they have a good relationship with their physician. This type of relationship involves two-way communication. However, this relationship can be influenced by the lack of specific training in communication, particularly in therapeutic education, the non-communication on the interest of biological assessments and regular follow-ups, the physician’s disinterest in the adherence of their patients to their medication, the overwork, and the lack of human resources.

### Limitations of the study

Our study had some limitations, including prevarication bias when collecting data on alcohol consumption and monthly income per household. Its cross-sectional design makes it difficult to document the causal relationship between exposure factors and non-adherence to hypertensive drugs that is measured at the same time.

In our study, it could seem that the way in which non-adherence to antihypertensive drugs was assessed is a limitation. According to the literature, several methods can be used to measure adherence to antihypertensive drugs. However, no method is ideal, and the preferential use of one over another essentially depends on the means available in daily practice. Given the limited financial resources of our study, we opted for an evaluation of medication adherence by interviewing hypertensive patients. Also, dividing the degree of adherence to antihypertensive drugs into two modalities could be considered a limitation preventing us from presenting an overview of the different types of non-adherence (e.g., non-persistence, intentional discontinuation, or unintentional discontinuation (e.g., forgetting). However, the objective of our study was essentially to measure the prevalence of non-adherence to drugs independently of the type of non-adherence.

## Conclusion

This study is very similar to the Meknes study but was carried out in a different region. Furthermore, the study methods were similar between the two sites, with the exception of this study in Marrakech, which evaluated self-reported depression.

The studies of Meknes and Marrakech found an important prevalence of non-adherence to hypertensive drugs. The study of Meknes found male sex, low monthly income, bad relationship with the healthcare system, and uncontrolled blood pressure as risk factors for non-adherence to antihypertensive drugs. In the study of Marrakech, non-adherence to hypertensive drugs is associated with stress, a lack of family support, the presence of depression symptoms, insufficient medical management of cardiovascular risk factors, uncontrolled blood pressure, and an unsatisfactory physician-patient relationship. Following these findings, we propose: (i) establishing continuing education sessions on therapeutic education for health professionals involved in the care of hypertensive patients; (ii) programming support groups; (iii) scheduling half-days for the care of hypertensive patients; (iv) ensuring the recruitment of health staff; and (v) establishing a performance-based remuneration system for health staff that will encourage them to take a greater interest in patient adherence.

## Supporting information

S1 Checklist Inclusivity in global research. (DOCX)
